# Supplementary figures and images for: Spatial and temporal dynamics of the bacterial community under experimental warming in field-grown wheat
Source: PeerJ. 2023 Jun 14;11:e15428. doi: 10.7717/peerj.15428 (PMC10276554; doi:10.7717/peerj.15428)

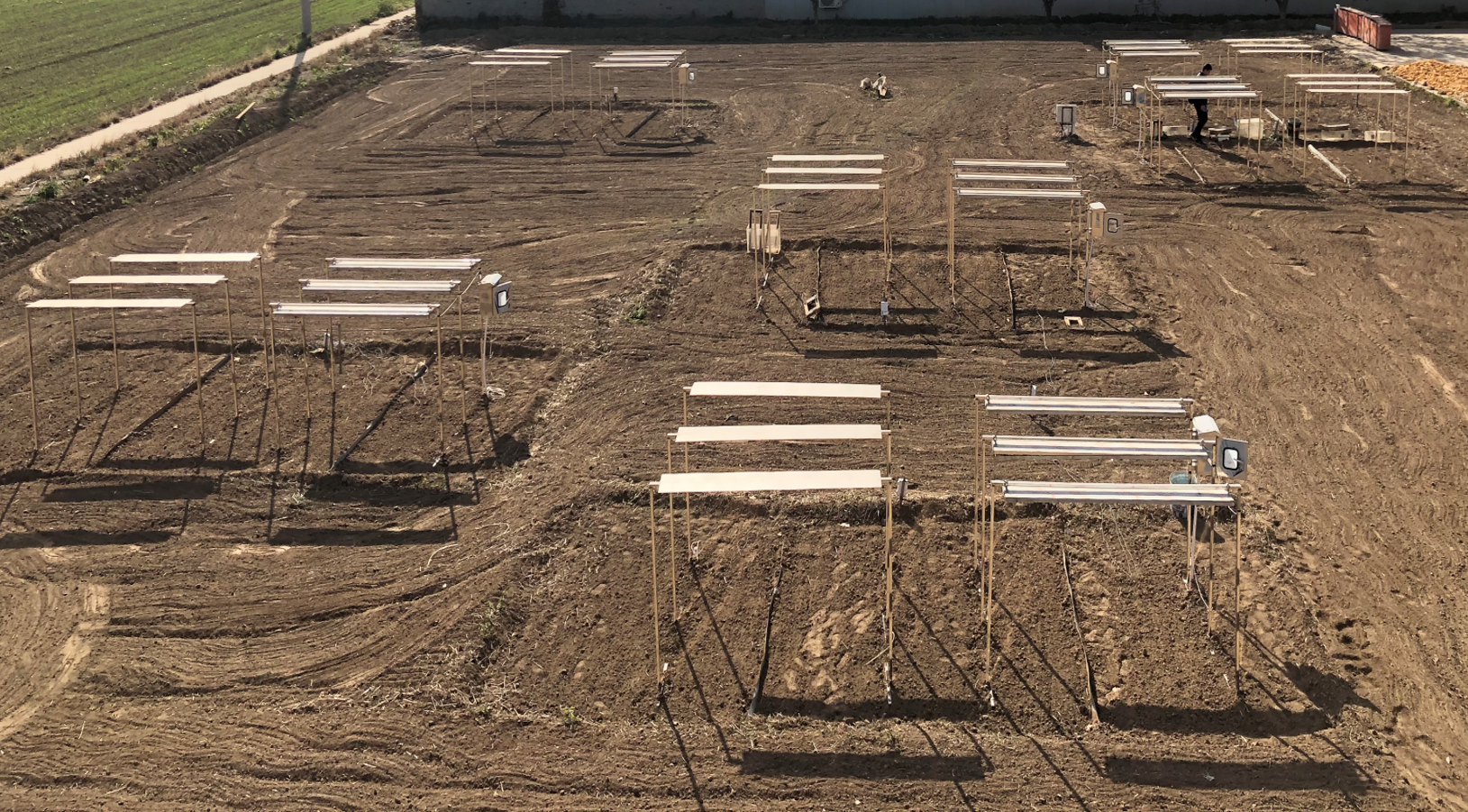

Supplement: Figure S1 [file peerj-11-15428-s001.png]

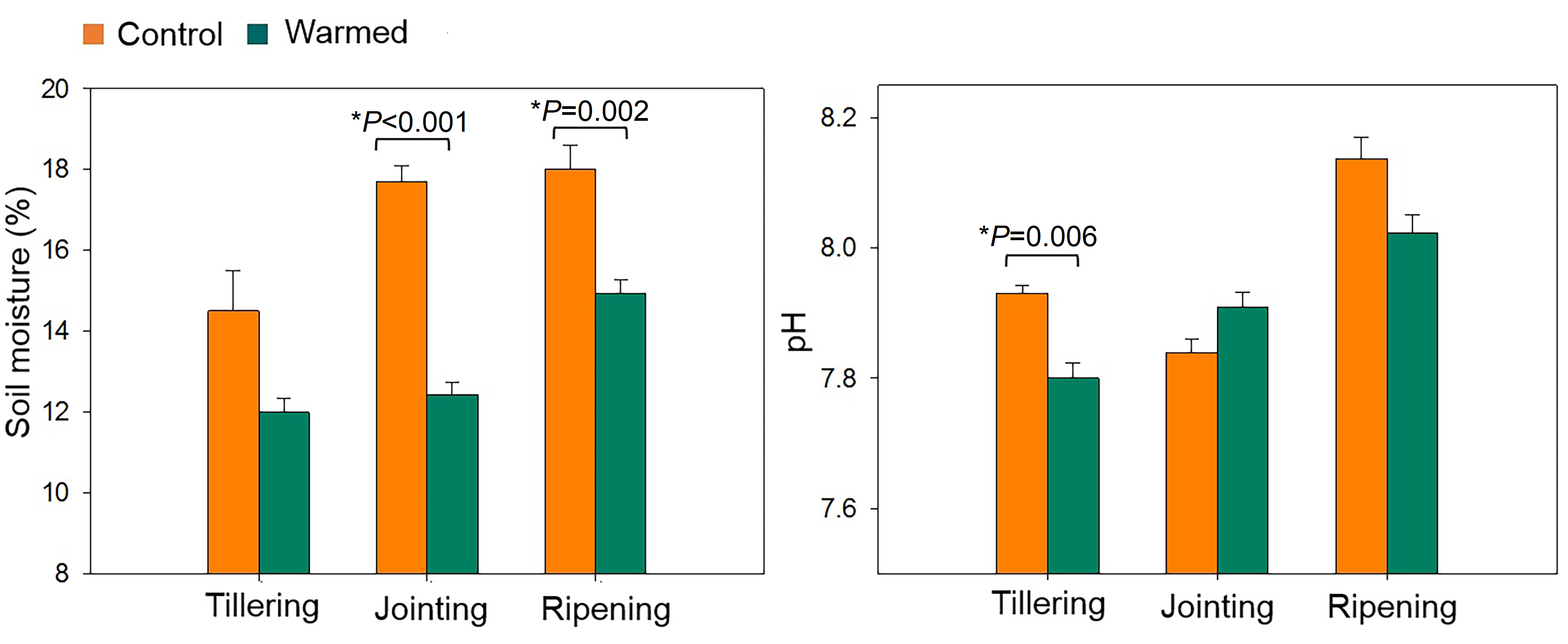

Supplement: Figure S2 [file peerj-11-15428-s002.png]

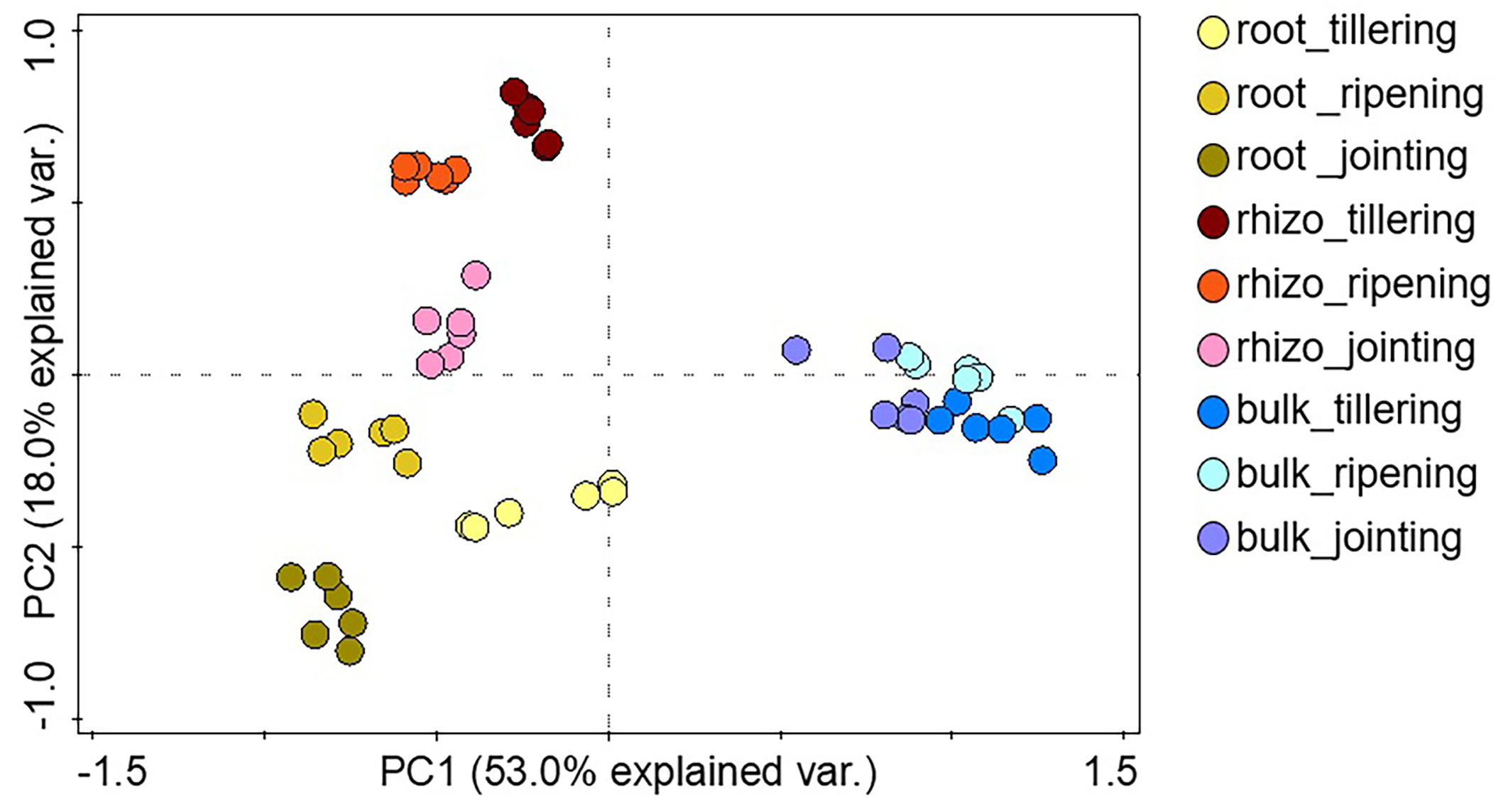

Supplement: Figure S3 [file peerj-11-15428-s003.png]

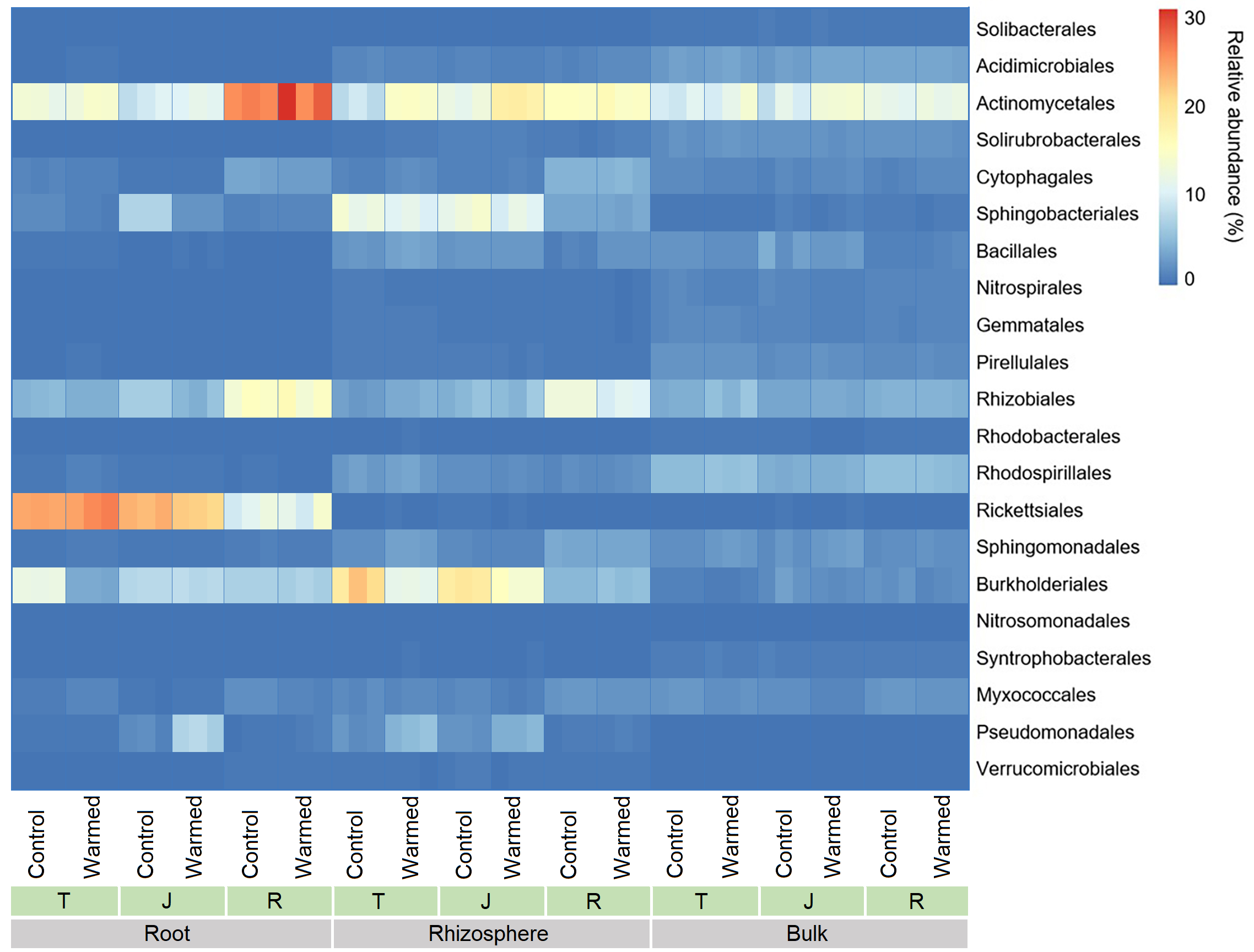

Supplement: Figure S4 [file peerj-11-15428-s004.png]

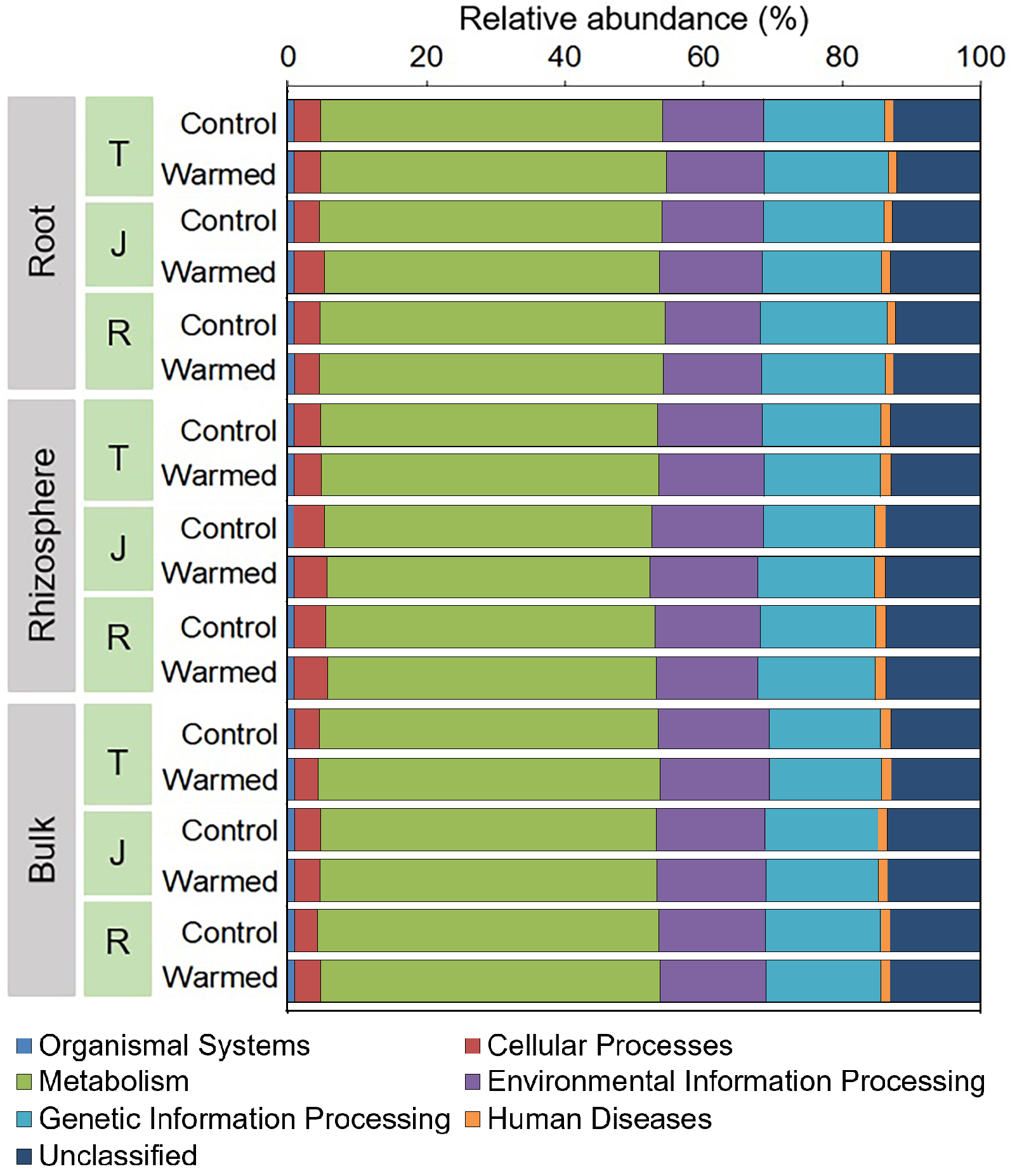

Supplement: Figure S5 [file peerj-11-15428-s005.png]

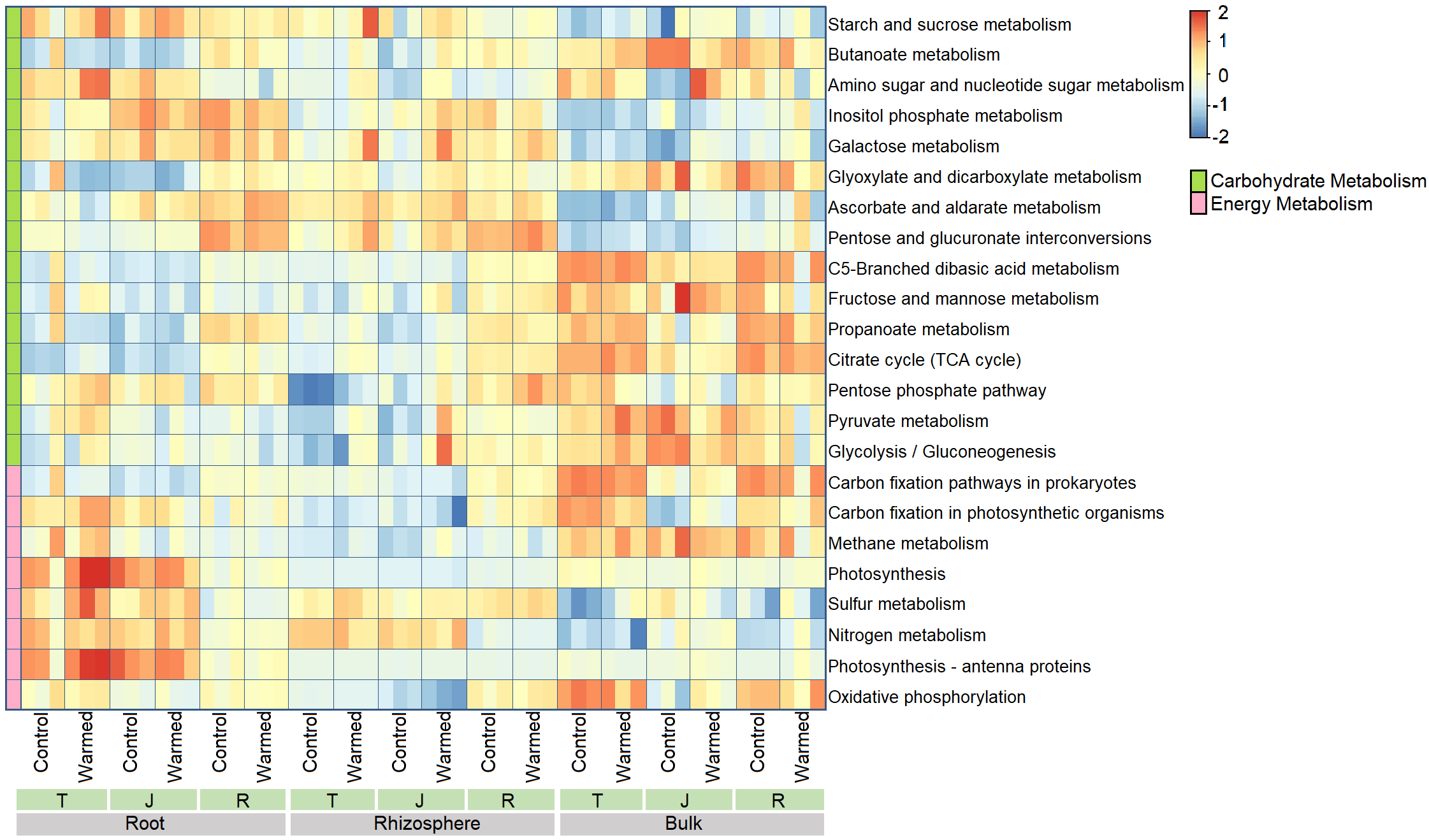

Supplement: Figure S6 [file peerj-11-15428-s006.png]
